# Mechanical Dentistry

**Published:** 1852-04

**Authors:** 


***Mechanical Dentistry.***—It was supposed by many, ten years ago, that mechanical dentistry had very nearly reached the *ne plus ultra* of perfection, but its progress since that time has been as rapid as at any former period, and in no branch of it, is improvement more manifest than in the manufacture of porcelain teeth in *blocks,* with artificial gums. The objections which existed a few years ago to dental substitutes of this sort, on account of their weight, and greater liability to be broken than single teeth, have, in a great measure, been removed by the manner in which they are now constructed and secured to the base. We recently had two double sets presented to us by Samuel Wardle, dentist, of Philadelphia, gotten up in a most beautiful style. The carving, the body, the enamel and gum, as well as the mounting, all are indicative of thorough knowledge and practical skill in this department of dentistry.

